# Heat Shock Response of the Active Microbiome From Perennial Cave Ice

**DOI:** 10.3389/fmicb.2021.809076

**Published:** 2022-03-10

**Authors:** Antonio Mondini, Muhammad Zohaib Anwar, Lea Ellegaard-Jensen, Paris Lavin, Carsten Suhr Jacobsen, Cristina Purcarea

**Affiliations:** ^1^Department of Microbiology, Institute of Biology, Bucharest, Romania; ^2^Department of Environmental Science, Aarhus University, RISØ Campus, Roskilde, Denmark; ^3^Center for Infectious Disease Genomics and One Health, Faculty of Health Sciences, Simon Fraser University, Burnaby, BC, Canada; ^4^Centre of Biotechnology and Bioengineering (CeBiB), Universidad de Antofagasta, Antofagasta, Chile; ^5^Departamento de Biotecnología, Facultad de Ciencias del Mar y Recursos Biológicos, Universidad de Antofagasta, Antofagasta, Chile

**Keywords:** ice caves, metatranscriptome, heat-shock response, active microbiome, microbial resilience, bioinformatics, meta-omics

## Abstract

Ice caves constitute the newly investigated frozen and secluded model habitats for evaluating the resilience of ice-entrapped microbiomes in response to climate changes. This survey identified the total and active prokaryotic and eukaryotic communities from millennium-old ice accumulated in Scarisoara cave (Romania) using Illumina shotgun sequencing of the ribosomal RNA (rRNA) and messenger RNA (mRNA)-based functional analysis of the metatranscriptome. Also, the response of active microbiome to heat shock treatment mimicking the environmental shift during ice melting was evaluated at both the taxonomic and metabolic levels. The putatively active microbial community was dominated by bacterial taxa belonging to Proteobacteria and Bacteroidetes, which are highly resilient to thermal variations, while the scarcely present archaea belonging to Methanomicrobia was majorly affected by heat shock. Among eukaryotes, the fungal rRNA community was shared between the resilient Chytridiomycota and Blastocladiomycota, and the more sensitive Ascomycota and Basidiomycota taxa. A complex microeukaryotic community highly represented by Tardigrada and Rotifera (Metazoa), Ciliophora and Cercozoa (Protozoa), and Chlorophyta (Plantae) was evidenced for the first time in this habitat. This community showed a quick reaction to heat shock, followed by a partial recovery after prolonged incubation at 4°C due to possible predation processes on the prokaryotic cluster. Analysis of mRNA differential gene expression revealed the presence of an active microbiome in the perennial ice from the Scarisoara cave and associated molecular mechanisms for coping with temperature variations by the upregulation of genes involved in enzyme recovery, energy storage, carbon and nitrogen regulation, and cell motility. This first report on the active microbiome embedded in perennial ice from caves and its response to temperature stress provided a glimpse into the impact of glaciers melting and the resilience mechanisms in this habitat, contributing to the knowledge on the functional role of active microbes in frozen environments and their response to climatic changes.

## Introduction

Ice can be considered as a storage matrix for microorganisms, representing a source of genomic diversity and a reservoir of new microbial species (Priscu et al., 1998; Miteva et al., 2009; Anesio and Laybourn-Parry, 2012; Anesio et al., 2017; Zhong et al., 2021). Recently, investigations of the microbial communities from a series of frozen environments have been performed, including permafrost (Schostag et al., 2019), Antarctic ice sheets (Abyzov et al., 2005; Weisleitner et al., 2019), Arctic ice (Ma et al., 2000), sea ice (Deming, 2002; Nichols, 2005), mountain glaciers (Garcia-Lopez et al., 2021), and subglacial lakes (Rogers et al., 2013). Meanwhile, very limited data regarding the microbiome embedded in perennial ice deposits accumulated in caves are available to help understand the resilience and ecological role of microbial communities from these secluded underground frozen habitats (Purcarea, 2018).

The Scarisoara ice cave (Romania) harbors the oldest and largest perennial ice block accumulated in a cave worldwide (Holmlund et al., 2005), representing a model habitat for studying paleoclimate processes (Perşoiu et al., 2011, 2017; Persoiu and Pazdur, 2011; Perşoiu and Onac, 2012; Rimbu et al., 2012) and the role and response to environmental stress factors of microbiomes preserved in underground ice from caves (Purcarea, 2018). The presence of ice-contained microorganisms in Scarisoara cave was first mentioned in the ice stalagmites formed in the Little Reserve area (Hillebrand-Voiculescu et al., 2013), followed by studies of the cultured/uncultured microbial communities from the perennial ice block (Hillebrand-Voiculescu et al., 2015) and the chronological distribution of cultured bacteria in ice layers up to 900 years old (Itcus et al., 2016). A series of psychrotrophic and psychrophilic bacterial strains were isolated from the 13,000-year-old ice core of this cave, confirming the microbial viability in this old icy habitat (Paun et al., 2021). Although the culturing method provided a step forward in microbial screening, scientists became aware of the limitations of culture-dependent techniques due to microbial uncultivability in describing the diversity of microbiomes (Hug et al., 2016). To overcome this problem, studies were conducted using denaturing gradient gel electrophoresis (DGGE) to unravel fungal diversity (Brad et al., 2018), while a more advanced sequence identification was achieved with the application of molecular techniques, including 454 pyrosequencing of the prokaryotic community (Itcus et al., 2018) and Illumina sequencing of the fungal communities along the 1,500-year-old ice based on ITS2 Illumina sequencing (Mondini et al., 2018).

These reports based on DNA sequencing provided information on the total communities, while no data on the metabolically active microbiome from this habitat have been provided so far. Recently, the total and potentially active bacterial communities from a 13,000-year-old ice core from Scarisoara have been determined using 16S ribosomal RNA (rRNA) Illumina sequencing (Paun et al., 2019), suggesting the existence of an active microbial community in this habitat. In this context, the current study focused on investigating the active microbiome from the Scarisoara cave ice using RNA Illumina shotgun sequencing and metatranscriptomic analysis of the total and active prokaryotic and eukaryotic microbial communities. Ice sample 900-O, previously collected from a sediment-rich ice layer accumulated 900 years ago in Scarisoara cave (Persoiu and Pazdur, 2011; Itcus et al., 2016), was selected considering the high diversity of prokaryotic and uncultured fungal communities found in this cave ice deposit (Itcus et al., 2018; Mondini et al., 2018).

Although reports on metatranscriptomes from frozen habitats revealed the presence of active microorganisms in ice (Rogers et al., 2013), the occurrence of an active microbiome in perennial ice from caves and its taxonomic and functional profiles are still unknown. Also, studies of the heat shock response of microbiomes from cold environments have been limited to soil (Schostag et al., 2019), while the mechanisms of coping with temperature shifts in the case of microbial communities from ice habitats are still not investigated.

In this context, the current survey represents the first investigation unraveling active prokaryotic and eukaryotic microbial communities in millennium-old underground perennial ice accumulated from caves based on total RNA [rRNA and messenger RAN (mRNA)] Illumina sequencing. Moreover, our data report the changes occurring at the taxonomic and metabolic levels in the total and active microbial communities in response to a 3-day cycle treatment followed by incubation at 4°C up to 14 days, which were done in order to understand the impact of glacier melting on the ice-embedded microbiome.

## Materials and Methods

### Site Description and Ice Sample Collection

**FIGURE 1:**
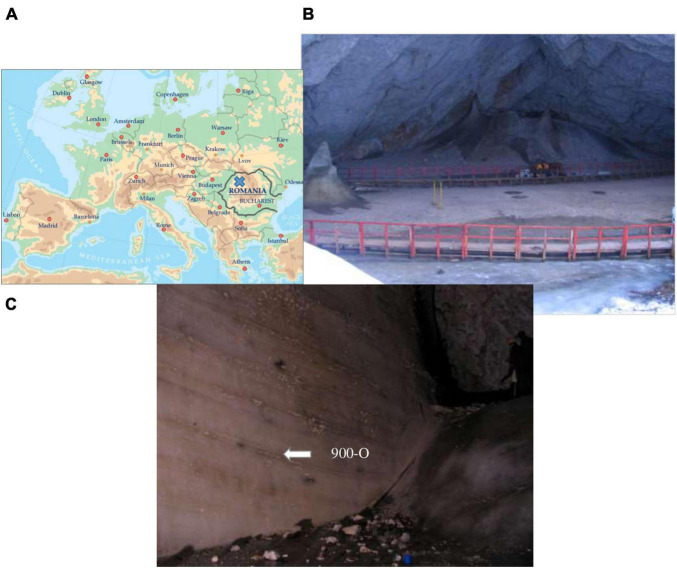
Scarisoara ice cave. **(A)** Map of the cave location. **(B)** The Great Hall area (photo by C. Purcarea). **(C)** The Little Reserve area (photo by C. Purcarea).

The Scarisoara ice cave located in the Apuseni Mountains (NW Romania) (Figure 1A) harbors one of the largest and oldest underground perennial ice blocks, ^14^C-dated to more than 10,500 years before present (BP) (Holmlund et al., 2005; Hubbard, 2017). The particular climate of the cave due to local temperate conditions and the large size of the entrance (Figure 1B) ensure underground constant negative temperatures, thus favoring the formation of a stable ice deposit (Racovita and Onac, 2000; Rimbu et al., 2012). The accumulated ice resulted from the annual freezing of precipitation and infiltration water constitute the alternating ice layers of organic-rich and clear ice deposits, with recent strata forming the floor of the Great Hall area and with an exposed side wall of up to 1,000 years BP ice in the Little Reserve sector (Figure 1C).

Previously (Hillebrand-Voiculescu et al., 2015; Itcus et al., 2016), ice samples of different ages were collected from the surface and side wall areas of the cave ice block to investigate the microbial diversity from this habitat. Among these, ice samples collected from the 900-year-old layer of high organic sediment content (Itcus et al., 2016) were used in this study (sample 900-O). The samples were obtained from the ice block side in the Little Reserve area (Figure 1C) by horizontal drilling using a modified PICO electric drill (Koci and Kuivinen, 1984) manufactured by Heavy Duties S.R.L (Cluj Napoca, Romania). Ice collection was carried out under aseptic conditions using 5-s flame sterilization of the auger and ice block surface before each step. The ice was harvested under aseptic conditions in 1-L sterile flasks, transported under stable frozen state monitored by an H-B Durac Bluetooth thermometer data logger (Sigma-Aldrich, Steinheim, Germany), and stored at −20°C until use (Itcus et al., 2016).

### Heat Shock Experimental Design

**FIGURE 2:**
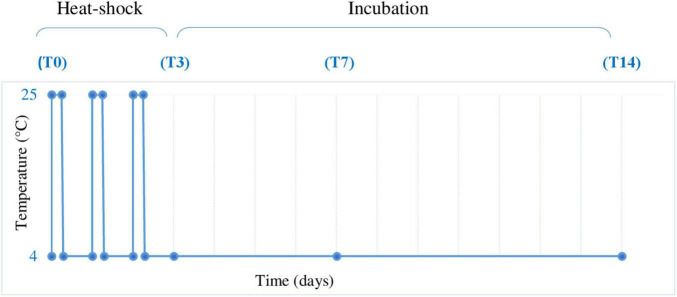
Heat shock experimental design. Melted ice samples stored at 4°C were submitted to 3 daily heat shock cycles (4–25°C) by incubation at 25°C for 8 h, followed by 4°C for 16 h and further incubation at 4°C for up to 14 days. The samples were collected before heat shock treatment (T0) and after 72 h (3 days, T3), 168 h (7 days, T7), and 336 h (14 days, T14), in triplicate, and analyzed as described in Section “Materials and Methods.”

The combined cave ice core samples were transferred in a 20-L autoclaved glass bottle under aseptic conditions using a microbiological biosafety cabinet to avoid contamination. After slow thawing at 4°C, the melted ice was equally distributed (1.7 L per sample) in 12 sterile 5-L bottles and submitted to thermal treatment (Figure 2). The process comprised three daily heat shock steps at 25°C for 8 h, followed by 16 h at 4°C and subsequent incubation at 4°C up to 14 days. Melted ice samples were analyzed prior to incubation (T0) and at 3 days (T3), 7 days (T7), and 14 days (T14) post-thermal stress (Figure 2). Three replicates were used for each analyzed step. In addition, a triplicate control was used by incubation of melted ice at 4°C for 14 days in the absence of thermal shock. After each step, the cells were collected by filtration and used for total RNA extraction.

### RNA Extraction, cDNA Library Preparation, and Illumina Shotgun Sequencing

The protocol for the preparation of RNA extraction and complementary DNA (cDNA) Illumina shotgun libraries (Mondini et al., 2019) was adapted from Schostag et al. (2019) and Bang-Andreasen et al. (2020). Triplicate ice samples were submitted to heat shock treatment and analyzed. After each heat shock step, the microbial cells from individual treated ice samples were immediately collected under aseptic conditions using a vacuum-driven stainless steel filtering system (Merck Millipore, Darmstadt, Germany) and sterile 0.22-μm microfiltration (MF) membranes (Merck Millipore, Darmstadt, Germany). Filters containing microbial biomass were placed in 15-mL sterile tubes with 2 ml G2 DNA/RNA Enhancer (Ampliqon, Odense, Denmark) and snap frozen for 15 s in liquid nitrogen. Total RNA extraction was carried out for each triplicate of the T0, T3, T7, and T14 samples using the RNeasy ^®^ PowerSoil ^®^ Total RNA Kit (Qiagen, Valencia, CA, United States) according to the manufacturer’s protocol. DNA contaminants were removed from the resulted RNA (50 μl) with the DNeasy Max ^®^ Kit (Qiagen, Valencia, CA, United States), and the purity and yield were determined by fluorometry using a Qubit 2.0 (Thermo Fisher Scientific, Roskilde, Denmark) in the presence of a Qubit DNA HS Assay Kit (Thermo Fisher Scientific, Roskilde, Denmark) and Qubit RNA HS Assay Kit, respectively. The RNA integrity number (RIN) was determined using a Bioanalyzer 2100 (Agilent Technologies, Glostrup, Denmark).

To perform the Illumina shotgun sequencing, a specific cDNA library was prepared for each of the 12 samples using 100 ng RNA and the NEBNext ^®^ Ultra II Directional RNA Library Prep Kit for Illumina ^®^ (New England Biolabs, Frankfurt am Main, Germany). The subsequent indexing process was carried out using the NEBNext Multiplex Oligos for Illumina and Index Primers Set 1 and the final cDNA library then purified using Sample Purification Beads (both from New England Biolabs, Frankfurt am Main, Germany). All the reactions were performed according to the manufacturer’s protocol in a dedicated PCR clean room. The cDNA library was quantified using a Qubit 2.0 spectrophotometer (Thermo Fisher Scientific, Roskilde, Denmark) and Qubit DNA HS Assay Kit (Qubit, New York, NY, United States). Library quantification was carried out using the KAPA Biosystems Library Quantification Kit for Illumina ^®^ Platforms (Merck, Søborg, Denmark) performing a series of 10-fold dilutions according to the manufacturer’s instructions. The final library quality control (QC) and the fragment size distribution were assessed using an Agilent Bioanalyzer 2100 and DNA Chips (Agilent Technologies, Glostrup, Denmark).

The resulting equimolar metatranscriptomic libraries obtained from each T0, T3, T7, and T14 triplicate were pooled and sequenced (150 bp paired-end) using a NextSeq 500/550 high-throughput kit v2.5 and the Illumina NextSeq platform (both from Illumina, San Diego, CA, United States) at the Department of Environmental Sciences, Aarhus University, Denmark.

### Bioinformatics and Statistical Analyses

The generated Illumina sequences (see *Data Availability Statement* for access code) were processed to assess the bioinformatics quality control and downstream analysis. Adapters and reads with an average quality score less than q20 and shorter than 60 bp were filtered using fastp (Chen et al., 2018). Reads were then sorted into small subunit (SSU) rRNA, large subunit (LSU) rRNA, and non-rRNA sequences using SortMeRNA v.2.1 (Kopylova et al., 2012).

SSU rRNA sequences were assembled into longer SSU rRNA contigs using MetaRib (Xue et al., 2020). Contigs were taxonomically classified using CREST (Lanzén et al., 2012), and the rRNA reads were mapped to the resulting MetaRib contigs using BWA (Li and Durbin, 2009), as performed in Bang-Andreasen et al. (2020), resulting in a table of taxonomically annotated read abundance across samples. In the case of the T3-3 sample (Table 1), the number of QC-rRNA reads was low (<10%), thus inconclusive for taxonomic assignment. Consequently, the metatranscriptome of this heat shock step was further represented only by duplicate samples.

**TABLE 1:** Paired-end read number and percentage of taxonomically assigned rRNA sequences from the Scarisoara ice microbiome submitted to heat shock.

Sample	Kingdom	Phyla	Class	Order	Family	Genus	Species
**Prokaryotes (paired-end reads)**							
T0-1	26,708,245	26,703,774	26,580,617	26,160,037	25,175,013	15,372,387	232,719
T0-2	27,836,047	27,831,490	27,705,786	27,265,799	26,228,364	16,033,779	236,871
T0-3	23,651,606	23,647,680	23,542,953	23,190,627	22,316,316	13,650,325	205,351
T3-1	28,282,664	28,282,278	28,213,325	28,011,200	27,349,501	15,875,298	250,887
T3-2	30,048,507	30,047,903	29,974,055	29,773,531	29,049,227	16,525,904	259,987
T7-1	28,597,318	28,596,970	28,524,035	28,237,593	27,581,953	15,576,993	275,193
T7-2	29,166,257	29,165,875	29,096,425	28,797,811	28,144,191	15,880,847	276,760
T7-3	28,056,523	28,056,226	27,986,377	27,828,392	27,184,407	14,488,376	234,245
T14-1	29,164,367	29,163,781	29,050,244	28,743,795	28,039,857	14,320,030	225,376
T14-2	23,308,066	23,307,516	23,252,628	23,055,747	22,457,392	11,849,106	176,860
T14-3	26,724,309	26,724,094	26,663,320	26,535,498	25,894,839	12,242,956	202289
Total	301,543,909	301,527,587	300,589,765	297,600,030	289,421,060	161,816,001	2,576,538
%	100	99.99	99.68	98.69	95.97	53.66	0.85
**Eukaryotes (paired-end reads)**							
T0-1	763,980	760,414	737,076	479,751	274,840	251,622	24,239
T0-2	871,593	866,740	831,435	502,818	272,033	245,268	26,419
T0-3	526,254	523,460	506,911	307,312	170,213	154,424	17,606
T3-1	86,733	86,430	81,668	75,446	39,425	32,040	6,868
T3-2	100,642	100,308	95,297	83,791	42,895	37,103	6,748
T7-1	275,448	275,341	251,498	247,971	92,595	56,769	6,426
T7-2	159,704	159,636	151,830	149,714	48,531	30,998	9,126
T7-3	555,313	555,133	545,029	534,363	102,128	91,387	23,573
T14-1	1,883,481	1,883,239	1,792,711	1,764,124	412,826	348,384	109,120
T14-2	985,865	985,791	911,617	895,093	235,039	205,225	47,561
T14-3	1,740,762	1,740,618	1,711,716	1,677,803	333,685	309,978	58,931
Total	7,949,775	7,937,110	7,616,788	6,718,186	2,024,210	1763,198	33,6617
%	100	99.84	95.81	84.5	25.46	22.17	4.23

CoMW (Anwar et al., 2019a,b) was used on a combined pool of non-ribosomal sequences from all samples. It uses trinity v.2.0.6 (Grabherr et al., 2011) for *de novo* assembly. CoMW filters non-coding RNA from the assembled contigs by aligning contigs to the Rfam database v.12.0 (Griffiths-Jones et al., 2003) with a significant *e*-value threshold of <10^–3^. CoMW also normalizes the contigs by removing those with relative expression lower than 1 out of the number of sequences in the dataset with the least number of sequences. The remaining contigs were then aligned against the M5nr protein database (Williams et al., 2013) and annotated using eggNOG annotation. Contigs were also aligned against specialist databases such as the Carbohydrate–Active Enzymes (CAZy) database (Cantarel et al., 2009) and the nitrogen cycling genes database (NCycDB) (Tu et al., 2019) to assess specific functions. Using CoMW, all alignments were filtered by keeping hits with a minimum *e*-value of 10^–5^ as the threshold. The scores indicated the abundance of number of reads from each sample assigned to groups of different functional genes from each database.

To identify the genes from eggNOG, CAZy, and NCycDB, gene families or functional subsystems that were significantly differentially expressed at different heat shock times, the DESeq2 (Love et al., 2014) module of the SarTools pipeline (Varet et al., 2016) was deployed using parametric mean–variance and independent filtering of false discoveries with the Benjamini–Hochberg procedure (*p* > 0.05) to adjust for type 1 error.

## Results and Discussion

### Community Composition and Thermal Response of the Potentially Active (rRNA) Ice Microbiome From Scarisoara Cave

Illumina shotgun sequencing of the total rRNA extracted from the untreated and thermal-treated cave ice microbiome generated a total of 414,992,189 paired-end reads assigned to potentially active bacterial, archaeal, and eukaryotic taxa.

Taxonomic assignment of the putatively active microbiome from the cave ice deposit (Table 1) led to the identification of a complex prokaryotic community composed of 42 bacterial phyla assigned to 103 classes and 182 orders, and 4 classes belonging to 1 archaeal phylum, in addition to a diverse eukaryotic community composed of 46 phyla, 76 classes, and 80 orders. The rRNA reads after each thermal treatment step could be assigned to a large extent to order (98.69%) and family (95.97%) ranks for prokaryotes and to class (95.81%) for eukaryotes, while only 0.85 and 4.23% of the corresponding communities could be identified at the species level (Table 1). Therefore, the profile variations of the heat-exposed ice microbiome were evaluated at the subsequent high taxon levels.

The initial cave ice microbial community (T0) was dominated by potentially active Bacteria (95.66 ± 0.76%), with a minor presence of Archaea (0.18 ± 0.02%) and low relative abundance of Eukarya (4.16 ± 0.43%) representatives (Table 2).

**TABLE 2:** Microbiome composition of the 900-O ice sample from the Scarisoara ice cave submitted to heat shock resulted from rRNA Illumina shotgun sequencing.

	Relative abundance (%)			
	T0	T3	T7	T14
Bacteria	95.66 ± 0.76	99.45 ± 0.02	98.68 ± 0.71	94.49 ± 1.17
Archaea	0.18 ± 0.02	0.02 ± 0.00	0.01 ± 0.00	0.02 ± 0.01
Eukaryotes	4.16 ± 0.43	0.53 ± 0.02	1.40 ± 0.72	5.49 ± 1.17
Algae	0.97 ± 0.20	0.07 ± 0.00	0.92 ± 0.69	4.58 ± 1.17
Metazoa	0.26 ± 0.10	0.05 ± 0.02	0.01 ± 0.01	0.01 ± 0.01
Fungi	0.24 ± 0.11	0.041 ± 0.00	0.02 ± 0.01	0.03 ± 0.01
Plantae	1.27 ± 0.23	0.19 ± 0.00	0.18 ± 0.01	0.22 ± 0.03
Protista	0.78 ± 0.12	0.15 ± 0.01	0.17 ± 0.01	0.63 ± 0.22
Protozoa	0.02 ± 0.00	0.01 ± 0.00	0.002 ± 0.00	0.005 ± 0.00

*Relative abundance was calculated based on the number of paired-end reads of the rRNA Illumina shotgun sequences of the untreated 900-O ice sample (T0), after 3-day heat shock treatment (T3), and after 7 days (T7) and 14 days (T14) incubation, as described in Section “Materials and Methods.” The average and standard deviation values were calculated for triplicate samples.*

Application of the heat shock cycles (T3) had a strong impact on the composition of the ice microbiome, leading to 9- and 8-fold decreases of the relative abundance of Archaea (0.02%) and eukaryotes (0.53 ± 0.02%), respectively, while the bacterial community appeared more stable prevailing (99.45 ± 0.02%) in the ice community exposed to temperature fluctuations (Table 2). Among the microbial eukaryotes, the strongest decline (12.5-fold) was observed in the case of the Metazoa taxon (from 0.25 to 0.02%). Thermal shock also induced an 8-fold decrease in relative content of both fungal (from 0.23 to 0.03%) and protozoan (from 0.8 to 0.01%) communities (Table 2).

Further incubation at 4°C for 2 weeks led to a partial recovery of the eukaryotic community, with relative content increases of up to 1.40 ± 0.72% (T7) and 5.49 ± 1.17% (T14) (Table 2). This trend appeared to be related to a slight decrease of bacterial presence after 7 days (98.68%) and 14 days (94.49%) incubation at 4°C post-treatment. Meanwhile, the composition of the archaeal taxon was very little affected, up to a relative abundance varying from 0.01% (T7) to 0.05 ± 0.01% (T14).

Overall, the response of the ice microbiome to heat shock treatment indicated a high resilience and a faster recovery of the bacterial taxon compared to the archaeal and eukaryotic taxa (Table 2).

### Heat Shock Response of the Ice Prokaryotic Community

**FIGURE 3:**
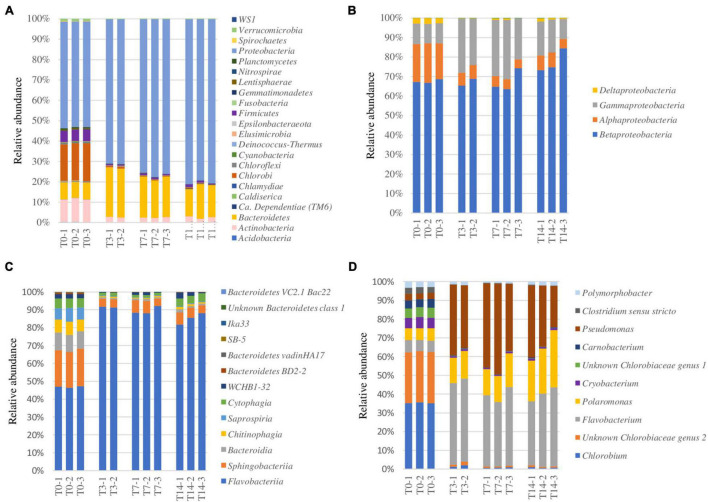
Heat shock effect on the potentially active bacterial taxa (rRNA) from the 900-O cave ice sample. **(A–D)** Relative abundance of bacterial phyla **(A)**, Proteobacteria classes **(B)**, Bacteroidetes classes **(C)**, and the most abundant 10 genera **(D)** of the ice microbiome before heat shock (*T0*) and after 3 days (*T3*), 7 days (*T7*), and 14 days (*T14*) of thermal treatments indicating variations in the bacterial community structure at specified taxonomic levels.

Microbial composition at the phylum level of the untreated (T0) cave ice potentially active (rRNA) bacteria indicated the presence of 53% Proteobacteria, 17.4% Chlorobi, 10.8% Actinobacteria, 8% Bacteroidetes, and 5% Firmicutes (Figure 3A). The higher relative abundance of Actinobacteria and the equal representation of Proteobacteria and Firmicutes in the 900-O ice sample compared to the ice core millennium-old strata (Paun et al., 2019) could be due to the different locations of the samples in the ice block. Major changes in the prokaryotic community composition were observed after the heat shock step (T3), resulting in a relative content decrease of Firmicutes by 10-fold (from 5 to 0.5%), Actinobacteria by 5-fold (from 10.6 to 2.6%), and Chlorobi by 5-fold (from 10.6 to 2.6%), while the representation of Proteobacteria and Bacteroidetes increased up to 71 and 27%, respectively (Figure 3A). A reduced effect was further observed after incubation at 4°C (T7 and T14), with a slight increase for Proteobacteria of up to 80%, whereas Bacteroidetes content showed a moderate decline to 15% after prolonged incubation (Figure 3A).

At the class level, Proteobacteria was mostly represented by Betaproteobacteria (68%), also containing Alphaproteobacteria (18%), Gammaproteobacteria (10%), and Deltaproteobacteria (3%) prior to heat shock exposure (T0) (Figure 3B). Within the Proteobacteria group, heat shock (T3) shaped the community by reducing the content of Alphaproteobacteria (6%) and increasing that of Gammaproteobacteria (25%), while maintaining an invariable (65%) Betaproteobacteria relative abundance. Prolonged incubation led to an increase of Betaproteobacteria (75%) alongside a reduction of Gammaproteobacteria (16%) and a slight increase in Alphaproteobacteria (7%) representation (Figure 3B).

The primarily represented classes of phylum Bacteroidetes (Figure 3C) from the untreated ice microbiome (T0) were Flavobacteriia (47%) and Sphingobacteriia (20%), with important representation of Bacteroidia (10%), Chitinophagia (8%), Saprospiria (7%), and Cytophagia (6%). The response to thermal treatment revealed a drastic drop of the relative abundance of most Bacteroidetes classes, while Flavobacteriia became the dominant group, constituting up to 90% of this phylum after heat shock (T3), and minor recovery of *Cytophagia* after 14 days (T14), indicating the high resilience of Flavobacteriia phylotypes to thermal stress (Figure 3C).

The Flavobacteriia, Betaproteobacteria, and Gammaproteobacteria classes responded positively to thermal treatment, outlining their advanced resilience coupled with their copiotrophic metabolism (Ho et al., 2017) and their quick response to a sudden nutrient increase (Fox et al., 2017). The associated increase of the relative abundance of these bacterial classes after thermal treatment might be a result of a multifactorial combination considering the high organic-rich composition of the 900-O sample (Itcus et al., 2016) and the metabolism of each specific taxon (Ramirez et al., 2012; Bastida et al., 2015). The reduced presence of Firmicutes phylotypes could be explained by their capacity to cope with environmental stressors by producing resistance cells, as proven by the presence of *Paenibacillus* sp., known for their ability to form endospores (Onyenwoke et al., 2004). The decrease of the relative content of Actinobacteria might have been due to their high sensitivity to temperature changes, as observed in the case of the soil microbiome (Schostag et al., 2019).

Among the 10 most abundant bacterial genera assigned in the T0 sample (Figure 3D), *Chlorobium* (35%) and *Chlorobiaceae genus 2* (28%) were the dominant ones, suggesting an important autotrophic activity. The initial microbiome structure also revealed an important (5–8%) presence of the genera *Polymorphobacter*, *Clostridium sensu stricto*, *Pseudomonas*, *Carnobacterium*, *Unknown Chlorobiaceae genus 1*, *Cryobacterium*, *Polaromonas*, and *Flavobacterium*. Heat shock treatment induced a redistribution of the major bacterial genera in the T3, T7, and T14 samples, leading to relative content increases from 6 to 15% (T7) and 20% (T14) of *Polaromonas* spp., a psychrophilic genus ubiquitous in glacial systems (Darcy et al., 2011), from 4% to 40–45% of *Pseudomonas* species widely present in Scarisoara ice block (Paun et al., 2019), and from 8 to 45% of *Flavobacterium* species commonly found in freshwater (Bernadet et al., 1996; Figure 3D).

The putatively active archaeal phylotypes identified in the cave ice samples were assigned to class Methanomicrobia (Euryarchaeota) and showed a strong drop in relative abundance after thermal treatment, from 0.16 ± 0.01% (T0) to 0.02 ± 0.00% (T3) and 0.01 ± 0.00% in both T7 and T14 samples. Other archaeal reads were identified only at *p*-values below the threshold (*p* < 0.005).

### Ice Fungal Community Response to Heat Shock

**FIGURE 4:**
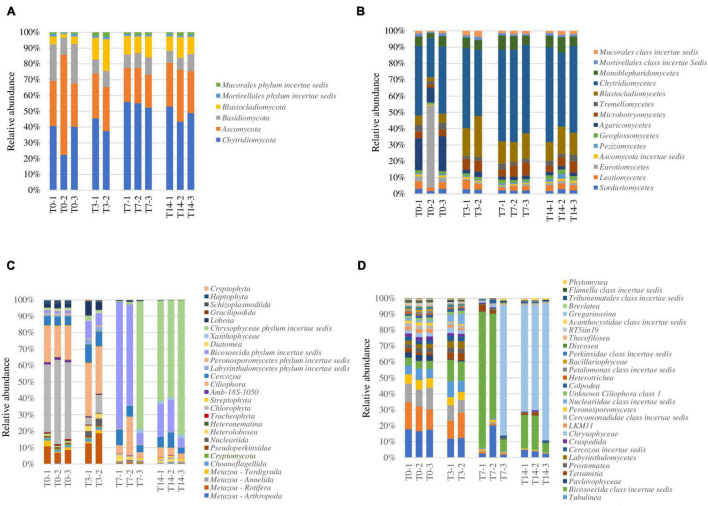
Analysis of the response of the potentially active eukaryotic community from the Scarisoara cave ice to thermal treatment before (*T0*) and after exposure to thermal stress (*T3*, *T7*, and *T14*), as described in Section “Materials and Methods.” **(A,B)** Relative fungal abundance at the phylum **(A)** and class **(B)** levels. **(C,D)** Relative abundance of eukaryotic phyla **(C)** and Protozoa classes **(D)**.

This rRNA-based survey constituting the first report on the potentially active fungal community structure in ice caves showed the presence of a complex community representing 0.23% of the Scarisoara ice microbiome (Table 2) assigned to 6 phyla and 14 classes (Figure 4).

At the phylum level (Figure 4A), the initial (T0) fungal community was shared among Chytridiomycota (40%), Ascomycota (30%), and Basidiomycota (20%), a highly spread taxon in frozen environments (Butinar et al., 2007), with a small (4%) relative content of Blastocladiomycota. The applied thermal treatment induced slight increases in the relative abundance of Chytridiomycota of up to 55% after 7 days of incubation (T7) and of Blastocladiomycota of up to 15% immediately after the 3-day treatment (T3), which was reduced to 10% after 14 days incubation at 4°C (T14). The relative abundance of both Basidiomycota and Ascomycota was reduced by heat shock (T3) to 8 and 20%, respectively, with preservation during the incubation at low temperature (T14) (Figure 4A).

At the class level (Figure 4B), the initial putatively active fungal community from cave ice (T0) was dominated by Chytridiomycetes (40%) and Agaricomycetes (20%). Eurotiomycetes was also highly represented in one of the T0 triplicates (50%), but only 5% in the other duplicates. The applied heat shock treatment (T3) shaped the fungal distribution by reducing the relative content of Agaricomycetes (4%) by 5-fold and increasing that of Chytridiomycetes phylotypes up to 50% (T3) and 58% (T14). Blastocladiomycetes, constituting 7% of the potentially active fungal community, also showed an increase in relative abundance of up to 20% (T3) immediately after heat shock, followed by a decline to 12% (T14). A similar trend was observed in the case of Microbotryomycetes, showing a slight increase in relative abundance (from 3 to 8%) in T3 that was stabilized after a longer incubation (T14) (Figure 4B).

The high relative abundance of Chytridiomycetes could be associated with the wide spread of this phylum in freshwater, marine habitats, and in soil (Money, 2016), according to their parasitic activity on planktonic algae (order Chytridiales) and their capacity to degrade chitin and keratin (McConnaughey, 2014). Moreover, this taxon is known as highly resistant to heat shock. The relative abundance of Blastocladiomycetes, a recently assigned phylum derived from Chytridiomycota (James et al., 2006), also increased (from 5 to 20%) after heat shock (T3) and remained at 11% after 7 days (T7) of incubation at 4°C. The resilience of these fungi, also present in freshwater, soil, and mud, could be related to their ability to decompose plant and animal debris and to parasitize arthropods (Money, 2016). Interestingly, the total fungal community from the 900-O ice sample based on DNA ITS2 Illumina sequencing (Mondini et al., 2018) indicated a dominant Ascomycota representation of uncultured taxa, unlike our data where Chytridiomycota was the main class found in the potentially active fungal community of this ice deposit.

At the genus level (Supplementary Figure 1), the potentially active fungal reads from the T0 sample could be assigned to the *Rhizophydium* (Chytridiomycota), *Geoglossum* (Ascomycota), and *Ochroconis* (Ascomycota) taxa, with relatively similar representation. *Rhizophydium* species belonging to *Rhysophydiales* are parasites of invertebrates, chytrids, and algae, assuming a possible role in the control of aquatic populations, and are also common in soil, primarily as saprobes, with a possible role in nutrient recycling (Powell, 1993; Nieves-Rivera, 2003; Ibelings et al., 2004; van der Wal et al., 2013; Burow et al., 2019). *Ochroconis* is a fungal genus reported in caves (Novakova, 2009; Martin-Sanchez et al., 2012b; Martinez-Avila et al., 2021), guano, and bats (Cunha et al., 2020). A few novel species were described in cave habitats, such as *Ochroconis anellii*, *Ochroconis lascauxensis*, and *Ochroconis anomala* (de Hoog and von Arx, 1973; Martin-Sanchez et al., 2012a). *Geoglossum* species are saprophytic fungi found in pastures and grassy forests (Kučera and Lizoň, 2012). Interestingly, no major effect was observed on the relative abundance of the identified fungal genera after heat shock (Supplementary Figure 1).

### Microeukaryotic Community Response to Heat Shock

The microeukaryotic group identified in the cave ice samples, representing the eukaryotic community mRNA without the fungal and plant assemblies, comprise 0.26% of the potentially active microbiome (T0) (Table 2). After the heat shock step (T3), the relative abundance of this microbial fraction was drastically reduced to 0.06%, followed by an increase of up to 5% after 14 days (T14).

Analysis of the taxonomic distribution of the microeukaryotes in the untreated T0 microbiome (Figure 4C) revealed the dominance of the phyla Tardigrada (4%) and Rotifera (10%), belonging to Metazoa, and the presence of more complex Protozoa group mainly assigned to the phyla Ciliophora (21%), Cercozoa (6%), and Bicosoecida (3%), in addition to Chlorophyta (Plantae) representatives (40%) (Figure 4D). In response to thermal treatment, the microeukaryotic group displayed an altered composition after each step, affecting various taxon compositions within the T0–T3, T3–T7, and T7–T14 intervals (Figure 4C).

Heat shock treatment (T3) reduced the relative content of Chlorophyta (5%) and increased those of the taxa belonging to Metazoa (15%), Ciliophora (30%), Cercozoa (10%), and Bicosoecida (12%). After a week of incubation at 4°C (T7), the microeukaryotic community was dominated by Bicosoecida (80%), with a severe representation loss in the Metazoa group (1%). The prolonged incubation (T14) of ice samples induced further changes in the distribution of microeukaryotes, leading to a clear dominance of Chrysophyceae (62%) and a reduction of the relative content of Bicosoecida to 20% (Figure 4C).

Potentially active Protozoa community from the 900-O untreated (T0) cave ice samples occupied 0.8% of the rRNA identified microbiome (Table 2). Assessment of Protozoa at the class level outlined a high diversity with virtually equal distributions of the classes Spirotrichea (19%), Oligohymenophorea (16%), and Litostomatea (10%) (Figure 4D). For this community, heat shock (T3) had no major effect, except for a slight drop of the relative abundance of Spirotrichea (12%) and Oligohymenophorea (11%) and a corresponding increase for class Bicosoecida (from 4 to 12%). Prolonged incubation outlined a diverse distribution coupled with varied class abundance between samples T7 and T14. After a week of incubation (T7), Bicosoecida was the dominant protozoan (80%), but after 14 days of incubation, its relative abundance dropped to 20% on behalf of Chrysophyceae (62%) representatives (Figure 4D).

The Metazoa group from the Scarisoara 900-O ice sample (T0) constituted 0.26% of the potentially active (rRNA) cave ice microbiome (Table 2), mainly composed of taxa belonging to the classes Bdelloidea (81.6 ± 3.5%) and Eutardigrada (14.3 ± 9.08%) (Supplementary Figure 2). While heat shock (T3) induced a 5-fold drop in the overall Metazoa content (Figure 4A), no major changes in the relative abundance of these classes were observed, where Bdelloidea represented up to 95% of the community after 7 and 14 days of incubation at 4°C (Supplementary Figure 2).

Prolonged incubation at a low temperature over 14 days delineated an increase of the relative abundance of the microeukaryotic community, highlighting the prospect of predation on the prokaryotic cluster (Figures 4C,D). Among the assigned reads, genera belonging to the Stramenopiles group, such as *Oikomonas*, *Spumella*, and *Bicosoeca*, displayed an increase in T7, with the highest representation after 14 days. The very high abundance of these genera could be explained considering a combination of the characteristics of Stramenopiles and the decline of the relative contents of prokaryotes in this sample. Stramenopiles are characterized by a smaller cell size compared to other protozoa (Ekelund and Rønn, 1994), corresponding to a faster duplication as colonizers, as proven in soil communities (Altenburger et al., 2010). The reduced occurrence of prokaryotes could be related to the ecological role of Protozoa and Metazoa as predators in bacterial communities (Rønn et al., 2012) enhanced in liquid environments. The latter assumption was corroborated by the observation of the correlated relative abundance of prokaryotes and protozoa (Table 2), suggesting an increase in the relative content of microeukaryotes based on the reduction of the bacterial community. In this view, the active participation of the Protozoa group in regulating bacterial abundance (Porter et al., 1985; Berninger et al., 1991) could play a role in the control of the fast growth of copiotrophic bacteria in organic-rich environments. Contrarily, representation of the Metazoa group could be restrained by the presence of Chytridiomycota, known for their parasitic endeavor on arthropods (Money, 2016).

### Heat Shock Impact on Gene Transcription (mRNA) of the Active Ice Microbiome

**FIGURE 5:**
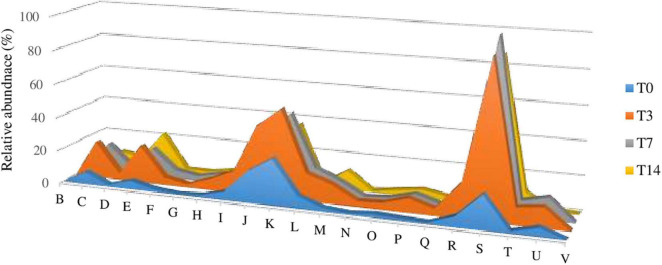
(A) Impact of heat shock on gene expression (mRNA) variations of the Scarisoara cave active ice microbiome. (B–V) Relative abundance values of the genes assigned by eggNOG gene annotations (level 2) involved in chromatin structure and dynamics (B); energy production and conversion (C); cell cycle control, cell division, and chromosome partitioning (D); amino acid transport and metabolism (E); nucleotide transport and metabolism (F); carbohydrate transport and metabolism (G); coenzyme transport and metabolism (H); lipid transport and metabolism (I); translation, ribosomal structure, and biogenesis (J); transcription (K); replication, recombination, and repair (L); cell wall/membrane/envelope biogenesis (M); cell motility (N); posttranslational modification, protein turnover, and chaperones (O); inorganic ion transport and metabolism (P); secondary metabolites biosynthesis, transport, and catabolism (Q); general function prediction only (R); function unknown (S); signal transduction mechanisms (T); intracellular trafficking, secretion, and vesicular transport (U); and defense mechanisms (V) calculated for the T0, T3, T7, and T14 untreated and treated samples, as described in Section “Materials and Methods.”

The mRNA analysis of the 900-O cave ice Illumina shotgun sequence highlighted the presence of an active microbial community in the Scarisoara ice cave, a groundbreaking result for this habitat. The gene transcriptional pattern of the T3, T7, and T14 thermal-treated ice communities compared to that of the untreated microbiome (T0) was modeled by both the increased temperature and prolonged incubation at 4°C, with the subsequent activation of different processes corroborated by the upregulation of specific gene clusters (Figure 5 and Supplementary Table 1).

**FIGURE 6:**
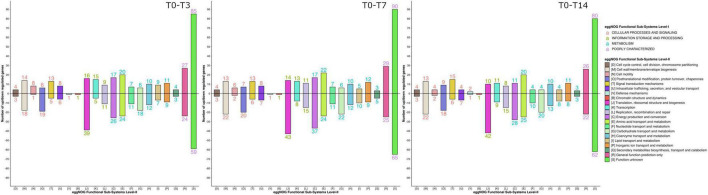
Gene expression shifts in response to the different thermal treatment steps. The profiles of the gene expression and number of upregulated and downregulated genes from different gene families (see Figure 5) during the T0–T3, T0–T7, and T7–T14 heat shock steps were determined as indicated in Section “Materials and Methods.”

Functional annotation using the eggNOG database revealed 639 differential functions. The impact of thermal shock and prolonged incubation on the metabolism of the active microbial community harbored in the Scarisoara millennium-old ice was deciphered by evaluating the gene expression profile of this microbiome belonging to different groups and after each thermal step (Figure 6 and Supplementary Table 1).

While the T0 sample showed a reduced general transcription, the T3 microbiome revealed the highest increase in mRNA overall transcripts (Table 3, Supplementary Figures 3, 4, and Supplementary Table 1). Thermal shock induced the transcription of genes involved in energy production and conversion (level 2, category C); amino acid transport and metabolism (level 2, category E); lipid transport and metabolism (level 2, category I); translation, ribosomal structure, and biogenesis (level 2, category J); transcription (level 2, category K); replication, recombination, and repair (level 2, category L); cell wall/membrane/envelope biogenesis (level 2, category M); signal transduction mechanisms (level 2, category T); and intracellular trafficking, secretion, and vesicular transport (level 2, category U). Moreover, the T7 sample displayed a comparable gene expression profile to that produced after 3 heat shock cycles (T3), but at a reduced level, whereas incubation for 14 days (T14) revealed a discrete reduction in the relative abundance of the C, J, K, L, and U genes.

**TABLE 3:** Differential functional gene expression of cave ice microbiome submitted to heat shock.

COG_ID	COG_Category_lvl_2	COG_Category_lvl_3	T0	T3	T7	T14
COG0513_1	[J] Translation, ribosomal structure, biogenesis	Superfamily II DNA and RNA helicases	745	1,408	1,376	–
COG0441	[J] Translation, ribosomal structure, biogenesis	Threonyl-tRNA synthetase	207	400	329	334
COG0202	[K] Transcription	DNA-directed RNA polymerase, alpha subunit/40-kDa subunit	1,048	1478	–	–
COG1959	[K] Transcription	Predicted transcriptional regulator	1,069	1,718	2,589	3,449
COG0114	[C] Energy production and conversion	Fumarase	52	368	202	171
COG0567	[C] Energy production and conversion	2-Oxoglutarate dehydrogenase complex, dehydrogenase (E1) component, and related enzymes	107	403	309	195
COG1894	[C] Energy production and conversion	NADH:ubiquinone oxidoreductase, NADH-binding (51 kDa) subunit	189	561	458	294
COG0045	[C] Energy production and conversion	Succinyl-CoA synthetase, beta subunit	173	450	311	–
NOG45042	[S] Function unknown	Phasin family protein	469	1,688	1,662	2,317
COG0683	[E] Amino acid transport and metabolism	ABC-type branched-chain amino acid transport systems, periplasmic component	169	432	–	347
COG0174	[E] Amino acid transport and metabolism	Glutamine synthetase	168	662	499	623
COG0459	[O] Posttranslational modification, protein turnover, chaperones	Chaperonin GroEL (HSP60 family)	2,718	788	663	710
COG0605	[P] Inorganic ion transport and metabolism	Superoxide dismutase	83	416	268	179
COG1344	[N] Cell motility	Flagellin and related hook-associated proteins	72	141		
COG2804_2	[N] Cell motility	Type II secretory pathway, ATPase PulE/Tfp pilus assembly pathway, ATPase PilB	12	145	85	45
COG0577	[V] Defense mechanisms	ABC-type antimicrobial peptide transport system, permease component	–	–	–	14
COG1619	[V] Defense mechanisms	Uncharacterized proteins, homologs of microcin C7 resistance protein MccF	–	–	–	28

*The mRNA reads of the untreated (T0) 900-O ice sample and after 3 (T3), 7 (T7), and 14 days (T14) of thermal treatment were assigned to different functional gene groups using the eggNOG database, as indicated in Section “Materials and Methods.”*

Analysis of the differential gene expression between the thermal treatment steps of various functional genes from the eggNOG database (Figure 6 and Table 3) showed an overall transcript increase substantiated by the upregulation of the genes involved in translation, ribosomal structure, transcription, replication, and repair (level 2, categories J, K, and L) processes, with the higher presence of transcripts for DNA and RNA helicases (level 3, category COG0513_1), threonyl-tRNA synthetase (level 3, category COG0441), DNA-directed RNA polymerase (level 3, category COG0202), and predicted transcriptional regulator (level 3, category COG1959). Increased transcriptional and translational activities were visible after thermal treatment and following 14 days of incubation (Figure 6 and Table 3).

A pronounced increase in the transcripts associated with the tricarboxylic acid (TCA) cycle (level 3, categories COG0114, COG0567, COG1894, and COG0045) was visible after the thermal shock step (T3), while a distinct reduction was evident after 7 and 14 days of incubation (Table 3). The increase in gene transcription involved in the TCA cycle substantiated the dominance of the copiotrophic taxa in the T3 and T7 samples, where the carbon sources are fully available after ice thawing, hence the need to release the stowed energy through oxidative processes. According to this hypothesis, the microbiome submitted to the 2-week thermal treatment (T14) mimicking putative natural daily phenomena after glacier melting displayed a declined transcription of these genes associated with reduced carbon sources for oxidative processes and a reduced presence of copiotrophic bacteria exposed to protozoa predation. Interestingly, the transcripts for the Phasin family proteins, such as polyhydroxyalkanoates (PHAs) (level 3, category NOG45042), were upregulated, in accordance with the energy storage by the microbial community after prolonged incubation at low temperatures. The increased energy storage could explain the dominance of copiotrophic bacterial cells also after a prolonged (14 days) heat shock incubation necessary for a composition shift in the copiotrophic–oligotrophic microbial community.

Transcripts involved in amino acid transport (level 3, category COG0683) and related to carbon and nitrogen metabolism using amino acids as intermediates (level 3, category COG0174) were also upregulated after the application of thermal treatment (Table 3). The transcripts for processes of amino acid transport were found associated with Proteobacteria (Suzuki et al., 2014), in accordance with the high relative abundance of this bacterial taxa in all To-T14 samples. Upregulation of the glutamine synthetase (GS) coding gene indicated the enhancement of nitrogen metabolism and the synthesis of glutamine (Hosie and Poole, 2001; Joo et al., 2018) of the ice microbiome submitted to thermal stress.

A direct response of the ice microbiome to temperature increase was also present after the ice thawing step, substantiated by the presence of transcripts involved in protein folding during thermal stress, such as GroEL (HSP60 family; level 3, category COG0459). Although the level of GroEL transcripts increased right after the ice thawing (T0), a significant decrease of this gene expression was observed (Table 3), which suggested a short response time for producing molecular chaperones to protect the microbial cells from the effects of high temperature. Another direct microbial response to the temperature increase consisted in the upregulation of transcripts coding for superoxide dismutase (level 3, category COG0605) in T3 samples (Table 3). This phenomenon, also described in *Escherichia coli*, indicated a direct correlation with the production of reactive oxygen species (ROS) in response to heat treatment (Marcén et al., 2017). Furthermore, the gene coding for alternative SigmaE factor, which controls the stress response at high temperatures (De Las Peñas et al., 1997), was highly upregulated after the thermal shock (T3), followed by a reduction in T7 and T14 samples. A slight increase in the relative abundance of cell motility genes (level 3, categories COG1344 and COG2804_2) was visible in T0 and T3 samples (Table 3), suggesting an increased motility associated with the water environment.

In addition, prolonged incubation for 14 days resulted in an increase in the defense mechanisms (level 3, categories COG0577 and COG1619) gene expression (Figure 6 and Table 3), suggesting the start of shortage of food sources and the competition among active microbes to outcompete the taxa utilizing similar substrates for community sustenance.

Elevated temperatures also accelerated fungal decomposition, resulting in increased carbon dioxide emission from the soil, which can lead to a faster temperature rise. A more detailed analysis could help in identifying the differential expression of genes involved in microbial respiration. Therefore, these genes could also be considered as potential markers for identifying specific microbial taxa adapted to higher temperatures that could contribute to global CO_2_ emissions as a primary driver of climate change.

Although development of the investigated system in the long run remains an open question, the short-term temperature changes (heat shock applied by environmental temperature shift on melted ice microbiome exposed on the soil surface) appeared to shape the active microbial community embedded in the Scarisoara cave ice. Extended investigations on long-term temperature variations will help deepen the knowledge on the microbial role in the global climate change.

## Conclusion

To date, this study provides the first evidence of an active microbiome and putatively active microeukaryotic taxa in perennial ice from caves, in addition to initial data on the thermal treatment response of the total and active ice-entrapped microbiomes using Illumina shotgun sequencing of rRNA and mRNA shift analysis. Temperature changes are known to disturb the microbial homeostasis alongside an altered taxon distribution, while little is known about the transcriptomic response to thermal stress. This overview of the structural and functional shifts in the ice microbial community induced by heat shock cycles and prolonged incubation contributed to increasing our understanding on changing environments and their ecological impacts due to ice melting.

The taxonomic profile of the potentially active microbiome from this icy environment was primarily modeled by heat shock, meanwhile revealing resilience mechanisms and specific functional responses to thermal stress. The ice community dominated by copiotrophic taxa was able to quickly use the carbon source released after ice thawing, with the Proteobacteria and Bacteroidetes taxa prevailing after thermal stress, unlike Archaea showing no recovery after incubation at a low temperature and the highly affected fungal community structure. Meanwhile, microeukaryotes exhibited fast recovery based on the putative predation process on bacteria. The resilience strategies of the active ice microbiome after heat shock exposure involved refolding, oxidative stress, and DNA synthesis-related gene upregulation. An increase in energy production and storage occurred during thermal stress, in accordance with the distribution of the dominant bacterial classes endowed with a copiotrophic lifestyle. Boosted carbon and nitrogen regulation and cellular motility appeared to respond to requirements for the water environment after ice melting, while the activation of defense mechanisms suggested a start of competition for similar food sources. The reported structural and functional modifications of the active microbial community from the Scarisoara cave ice due to thermal variations offered a glimpse of the environmental impacts of climate change leading to glacier retreat. In-depth analyses of the reconstructed metagenomes could contribute to unraveling the evolutionary patterns of metabolic pathways in this secluded cold habitat, subtle molecular adaptation mechanisms to icy environments, and novel cold-active biomolecules of applicative potential. Meanwhile, this first report on a critical shift of the active microbiomes from melting ice habitats represents a warning for possible major changes of the environmental biogeochemistry associated with late accelerated ice loss.

## Data Availability Statement

The sequencing datasets presented in this study are publicly available at Sequence Read Archive (SRA) under the BioProject ID PRJNA777283 (https://www.ncbi.nlm.nih.gov/bioproject/PRJNA777283).

## Author Contributions

AM and CP wrote the manuscript. AM performed the sample filtration, heat shock experiment, RNA extraction, and cDNA library preparation. LE-J and CJ designed the thermal treatment experiment and carried out the Illumina shotgun sequencing with contributions from AM and MZA. MZA performed the bioinformatics and statistical analyses. AM and PL contributed to the sequence analyses. CP performed the experimental design and coordinated the project. All authors contributed to data interpretation and revised the manuscript.

## Conflict of Interest

The authors declare that the research was conducted in the absence of any commercial or financial relationships that could be construed as a potential conflict of interest.

## Publisher’s Note

All claims expressed in this article are solely those of the authors and do not necessarily represent those of their affiliated organizations, or those of the publisher, the editors and the reviewers. Any product that may be evaluated in this article, or claim that may be made by its manufacturer, is not guaranteed or endorsed by the publisher.
