# Supplementary material for: Heat Shock Response of the Active Microbiome From Perennial Cave Ice
Source: Front Microbiol. 2022 Mar 10;12:809076. doi: 10.3389/fmicb.2021.809076 (PMC8960993; doi:10.3389/fmicb.2021.809076)
Supplement: Supplementary file 1 [file Data_Sheet_1.PDF]

Supplementary Material

Heat shock response of the active microbiome from perennial cave ice

Antonio Mondini[1#], Muhammad Zohaib Anwar[2,3#], Lea Ellegaard-Jensen[2], Paris Lavin[4,5], Carsten Suhr Jacobsen[2], Cristina Purcarea[1]

# Authors with equal contribution

*** Correspondence:** Cristina Purcarea, cristina.purcarea@ibiol.ro

Supplementary Table S1. Differential gene expression in Scarisoara active microbiome submitted to thermal treatment.

Differential Expression Analysis							
**Reference**	**Target**	eggNOG		CAZy		NCycDB	
		down	*up*	*down*	*up*	*down*	*up*
T0	T3	302	289	319	444	45	59
T0	T7	348	290	342	443	48	83
T0	T14	325	253	283	359	42	49
T3	T7	4	18	24	37	5	1
T3	T14	12	53	38	143	10	25
T7	T14	18	23	15	70	7	12

The number of upregulated and downregulated genes between each steps of the thermal treatment was calculated using eggNOG, CAZy and NCyc databases for total metatranscriptome, carbohydrates and nitrogen metabolism, respectively. (T0): untreated sample; (T3) after 3-daily heat-shock cycles; (T7) after incubation at 4ºC up to 7 days; (T14) after incubation at 4ºC up to 14 days.

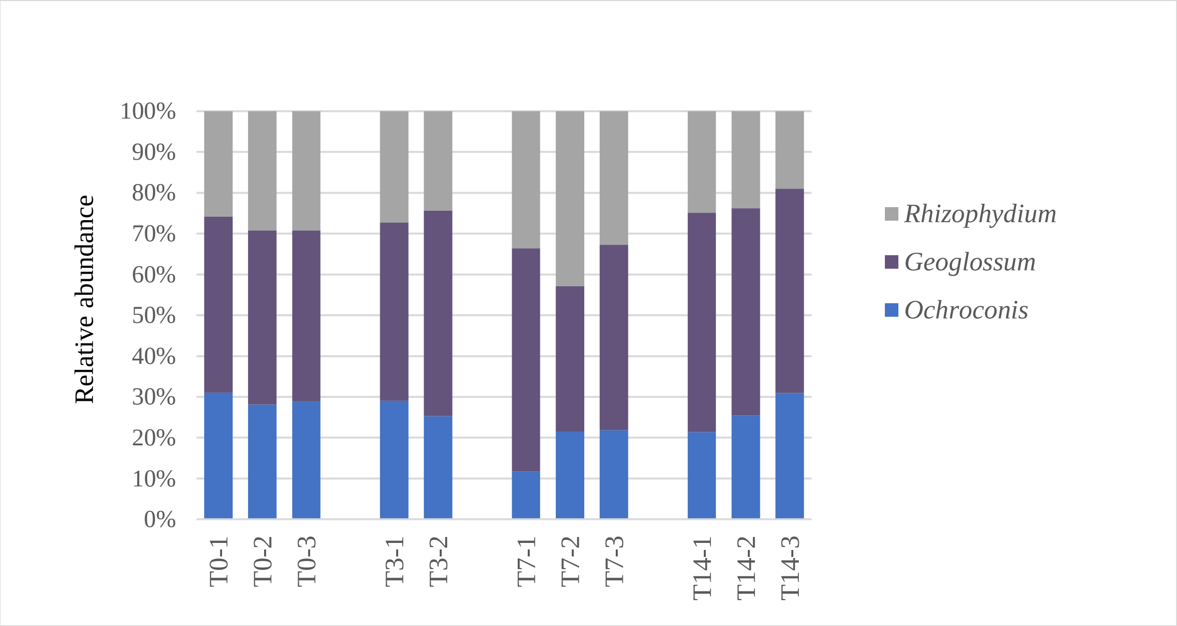

Supplementary Figure S1. Heat-shock response of the potentially active fungal genera from Scarisoara cave ice. Relative abundance before (T0) and after exposure to thermal stress (T3, T7, T14) was analyzed as described in Methods.

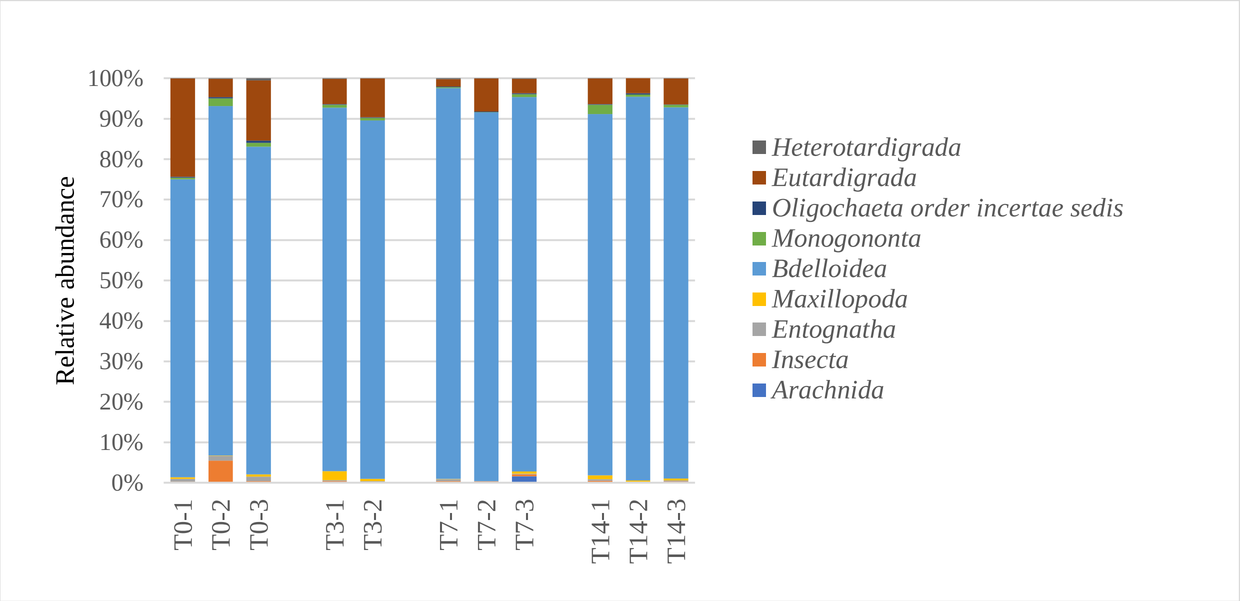

Supplementary Figure S2. Heat shock response of potentially active (rRNA) Metazoa classes from Scarisoara cave ice. The relative abundance was analyzed before (T0) and after (T3, T7, T14) thermal treatment as described in Methods.

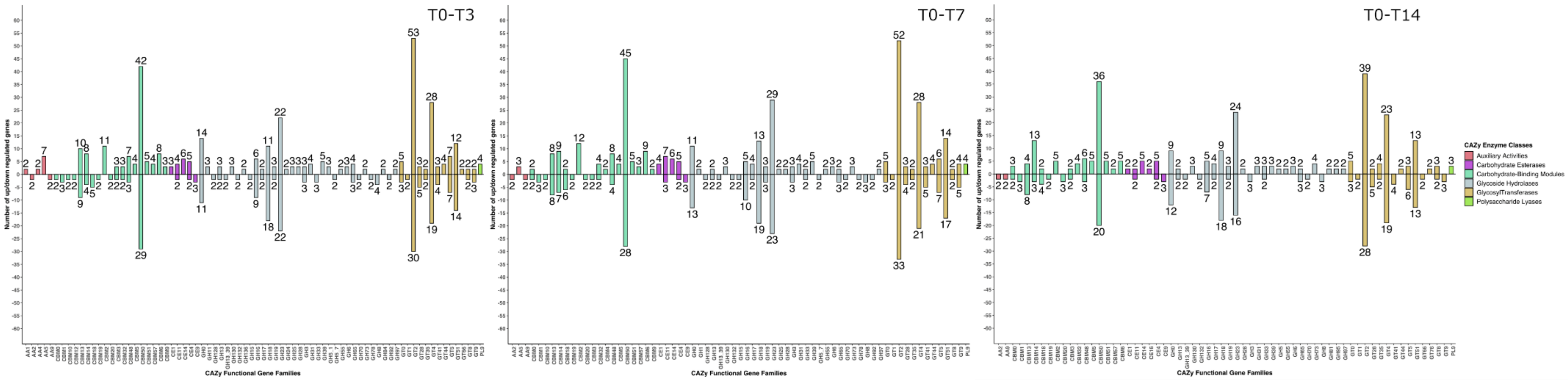

Supplementary Figure S3. Heat-shock response of the carbon metabolism of the active microbiome from Scarisoara cave ice. The upregulated and downregulated number of genes within different genes families and enzyme classes from carbon (CAZy) pathways during T0-T3, T0-T7 and T7-T14 heat-shock steps were calculated as indicated in Methods section.

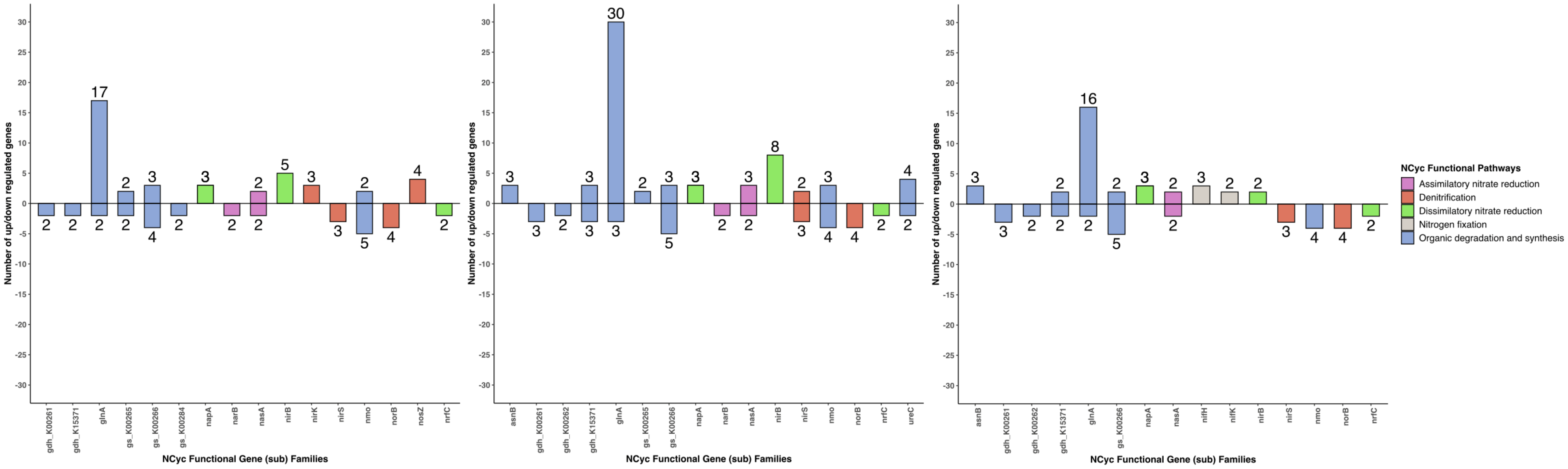

Supplementary Figure S4. Heat-shock response of the nitrogen metabolism of the active microbiome from Scarisoara cave ice. The upregulated and downregulated number of genes within different genes families and pathways from Nitrogen (NCyc) during T0-T3, T0-T7 and T7-T14 heat-shock steps were calculated as indicated in Methods section.
